# Perforated ileal phytobezoar revealed a MALT lymphoma

**DOI:** 10.11604/pamj.2016.25.16.10361

**Published:** 2016-09-20

**Authors:** Adriá Rosat, Juan Manuel Sánchez

**Affiliations:** 1 Department of General Surgery, Hospital Universitario Nuestra Señora de Candelaria, Ctra Del Rosario 145, 38010 Sta Cruz de Tenerife, Spain

**Keywords:** Phytobezoar, MALT lymphoma, ileum, perforation

## Image in medicine

A 45-year-old woman was under study for one year of abdominal pain associated with 4kg weight loss and chronic constipation. An entero magnetic resonance found a mass of 8x10cm on medium ileum suggesting a phytobezoar, with no proximal bowel dilatation. The woman was scheduled for programmed resection. Two weeks after, she presented on our emergency department with an acute abdomen. Exploratory laparotomy showed a bowel perforation of the phytobezoar, and a segmental resection was performed. No more bezoars were found on exploration. She did well after surgery and was discharged home on 6th post-operative day. Pathology revealed a MALT Lymphoma (cells were CD20-positive B-lymphocytes that co-expressed BCL-2 and were negative for CD3, CD5, CD10, CD23, BCL-6 and cyclin D1 on immunohistochemical studies). The overall proliferation index was low with Ki-67 immunoreactivity in approximately 10% of cells. The bone marrow biopsy showed no evidence of involvement by lymphoma. The patient started therapy with rituximab infusions (monoclonal antibody against CD20). In two years follow up she remains asymptomatic and without recurrence. Primary ileal MALT lymphoma is rare, and has not been associated with a specific infectious disease. Bezoar is an unusual cause of small bowel obstruction accounting for 0.4-4% of all mechanical bowel obstruction. The common site of obstruction is terminal ileum. We believe that the primary MALT lymphoma caused a small mechanic obstacle that made alimentary fibers to accumulate and cause the phytobezoar. The perforation required a segmental resection, which finally led to the diagnostic of the MALT lymphoma.

**Figure 1:**
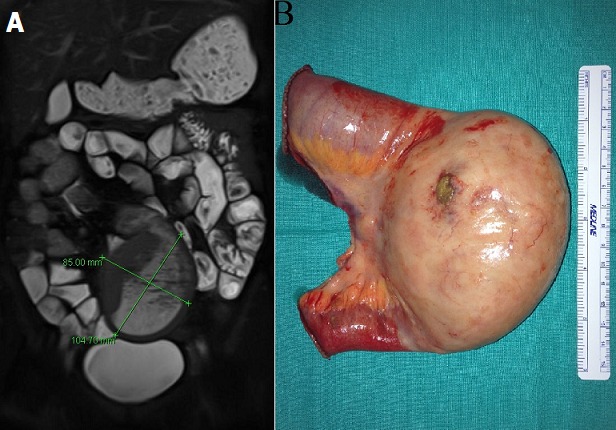
(A) entero magnetic resonance showing a solid mass of 8x10cm on medium ileum without proximal bowel dilatation, suggesting a bezoar; (B) piece of resection showing the perforation

